# A Treatise on Therapeutics

**Published:** 1879-06

**Authors:** 


A Treatise on Therapeutics, Comprising Materia Medica and Toxicology, with especial reference to the Application of the Physiological Action of Drugs to Clinical Medicine. By H. C. Wood, Jr., M.D., Prof. of Mat. Med. and Therapeutics, and Clinical Professor of Diseases of the Nervous System, in the University of Pennsylvania. Third edition, revised and enlarged. J. B. Lippincott & Co., Philadelphia. 1879.

In this work the effort has been to illustrate the physiological action of medicines with a comparative disregard to their "empirical or clinical" results. The idea that "we must discover what influence a drug exerts when put into the body of a patient, before we can use it rationally,"

is founded on "good hard sense," and is far preferable and more satisfactory than the routine course of a strict clinical therapeusis. This doctrine is destined to triumph, and the day will come when printed formularies will not be *countenanced*, but direct physiological action will, we hope, be universally sought.

The classification adopted in the work, we are sorry to say, is not founded entirely on this principle, and is, therefore, as well as all others yet made, subject to objection. The class of tonics, for example, is correctly said by the author "to restore lost tone," but the organs to which this lost tone is given, are not definitely pointed out by their names, as in our opinion they should be.

We are pleased to see, however, that Prof. Wood is leading off in the proper direction. The idea of studying the physiological action of remedies, promulgated by his relative, the late Dr. Geo. B. Wood, more than the fourth of a century ago, has been elaborated by the author before us.

